# MXene-Based Lithium-Ion Anodes: An Outlook on Computational Predictions and Experimental Validation

**DOI:** 10.1021/acs.energyfuels.6c02894

**Published:** 2026-09-30

**Authors:** Abhinav Ernam, Eugenie Pranada, Bright Ngozichukwu, Abdoulaye Djire

**Affiliations:** † Artie McFerrin Department of Chemical Engineering, 14736 Texas A&M University, College Station, Texas 77843, United States; ‡ Department of Materials Science and Engineering, Texas A&M University, College Station, Texas 77843, United States

## Abstract

First-principles studies have produced extensive predictions of MXene-anode capacities, voltages, and diffusion barriers over the past decade. Yet, a gap remains between these predicted metrics and the performance observed in operating cells. We suggest that this gap reflects not a limitation of computation itself but the absence of a sustained feedback loop between computational and experimental work. Computational predictions are often communicated and cited as performance forecasts even though they describe idealized systems (bare or uniformly terminated surfaces, equilibrium conditions, and the absence of electrolyte, interphase, and cycling history) that differ from the environment of an operating anode. Focusing on MXene lithium-ion anodes, we examine where predicted and measured capacities diverge, distinguishing predictions that are inaccurate from predictions that are simply applied beyond the conditions they were constructed to describe. We then consider the physics that standard protocols are not designed to capture and outline three coordinated directions for reconnecting computation to operating reality: coordinated theory-experiment studies as a structural foundation, machine-learning interatomic potentials to reach the relevant time scales, and explicit electrolyte modeling to recover interfacial energetics often omitted from vacuum-reference calculations. We propose that the progress of MXene-anode research be measured by how closely computation and experiment inform each other.

## Graphical abstract

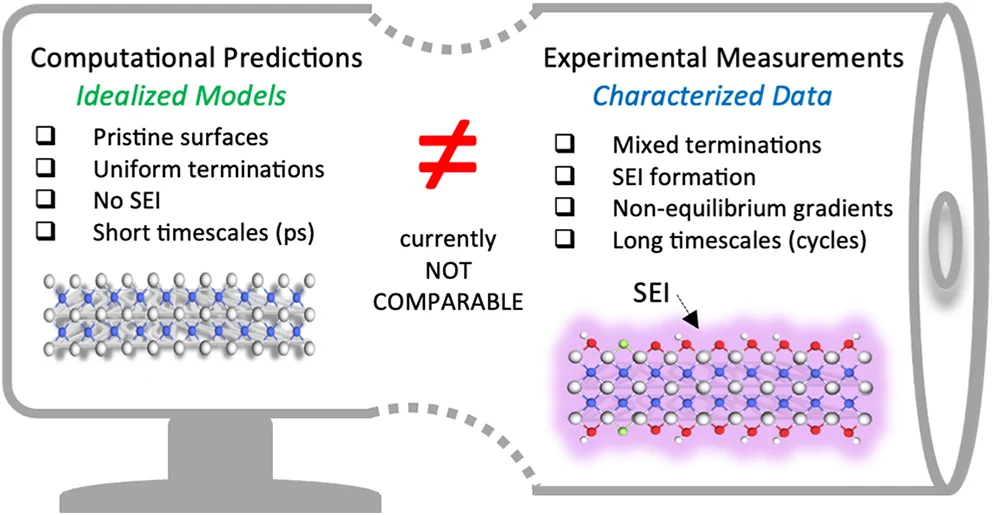

## 1 Introduction

Lithium-ion batteries (LIBs) underpin portable electronics, electric vehicles, and grid-scale energy storage, and the demand for higher energy density and longer cycle life continues to outpace what current chemistries deliver.^1^ Among the components limiting further progress, the anode has become a central focus. Graphite anodes are near their theoretical capacity limits, shifting focus to high-capacity alternatives like silicon and lithium metal. However, commercial adoption remains constrained by severe volume expansion, dendrite formation, and interphase instability.^2^

Two-dimensional transition metal carbides and nitrides, known as MXenes, offer a combination of high electronic conductivity (up to 20,000 S cm^–1^ for Ti_3_C_2_T_*x*_) that conventional anode materials cannot match.^3^ Their atomically defined layered structures, tunable surface terminations, and capacity to accommodate Li^+^ within interlayer regions have made them the subject of intensive computational and experimental investigation over the past decade.^4,5^ First-principles studies in particular have produced a steady stream of predicted capacities, voltage profiles, and diffusion barriers across the M_n+1_X_n_T_*x*_ family (where M is an early transition metal, X is carbon and/or nitrogen, T_*x*_ is surface terminations, and n is layer number) generating design heuristics that the field now widely cites: oxygen terminations favor lithium capacity, lighter transition metals yield higher gravimetric capacity, and metallic density of states at the Fermi level is preserved across most functionalizations.^5–7^

Yet, the capacity values most often cited for MXene anodes stand well above what operating cells deliver. In specific well-studied cases, most notably Ti_3_C_2_T_*x*_, theoretical capacities cited at the upper end of computational predictions exceed measured operating capacities by factors of three or four.^8,9^ The discrepancy likely arises from a combination of assumptions about surface termination, electrolyte environment, and cell conditions that the calculations embed but may not entirely mirror experimental conditions.^10,11^ Reported capacities for similar compositions often vary across studies due to differences in experimental protocols that may not be fully detailed. Consequently, the gap between predicted and measured performance stems from both sides: idealized models are compared to experimental data from conditions they were not designed to simulate, while the experimental data itself can be difficult to compare across different laboratories (Figure 1).

**Figure 1:**
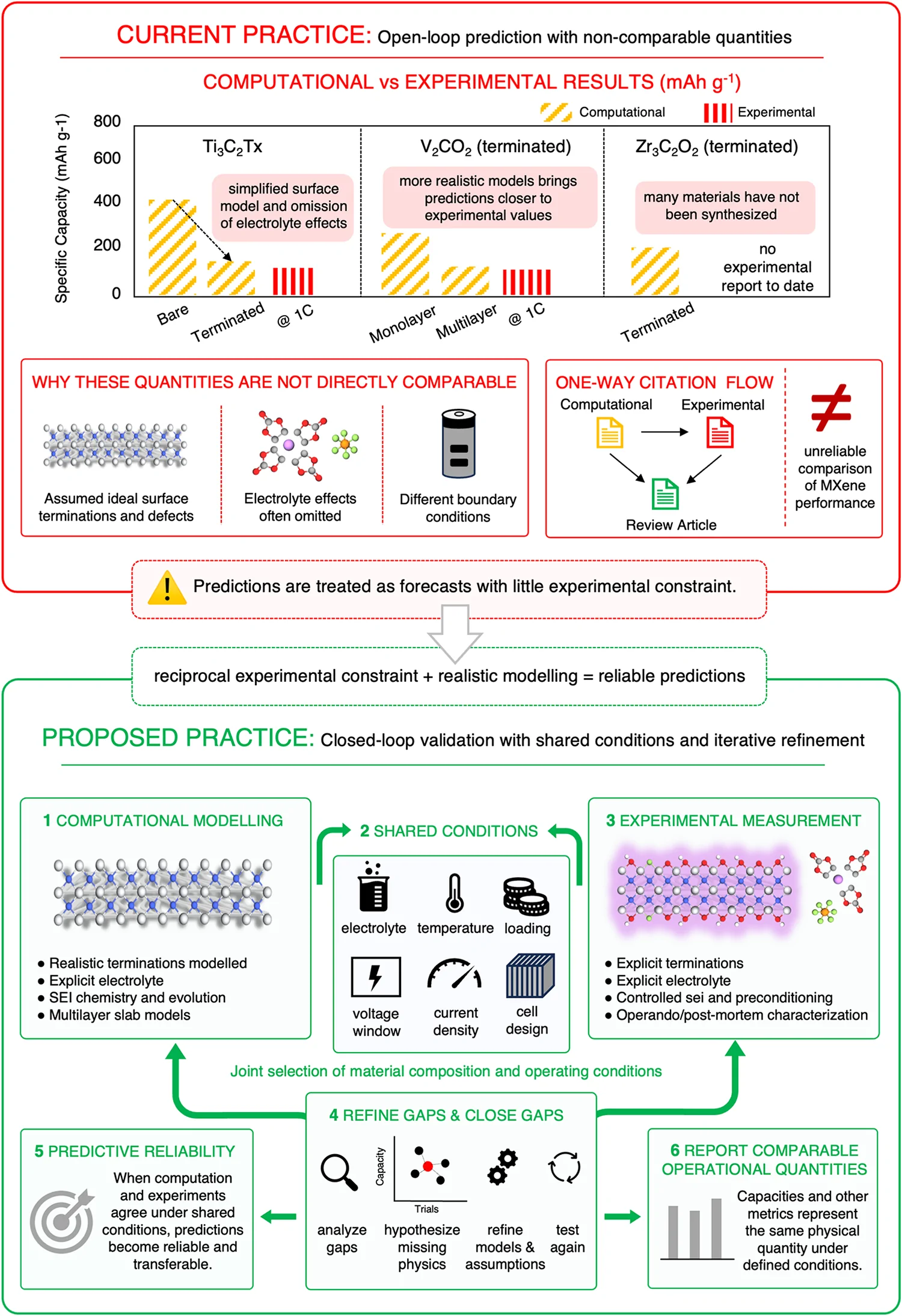
Conceptual shift from isolated current practices to a mutually constrained computational-experimental framework.

The persistent gap between predicted and demonstrated MXene-anode performance reflects less a limitation of computation than the limited integration between computational and experimental work. Both communities have advanced rapidly; what remains underdeveloped is the systematic integration of computational and experimental work. In adjacent battery-materials fields, computational predictions have proven informative when constrained by experimental boundary conditions and have been refined under iterative comparison with measurement.^12,13^ MXene-anode research is yet to build this practice. What follows examines where the disconnect originates, what current computational protocols can and cannot capture, and what would be required for computation to become reliably predictive of MXene-anode behavior in operating cells. The question addressed is direct: what does it take for computational predictions of MXene-anode performance to become reliably predictive of experimental behavior?

## 2 The Predictive Gap in MXene-Anode Research

MXene-anode research is a particularly informative system for examining how computational predictions and experimental measurements relate because computational and experimental studies of MXene anodes have accumulated rapidly over the same period, often addressing the same compositions.^6^ First-principles studies have generated capacity, voltage, and diffusion-barrier predictions across most members of the MXene family, and experimental work has measured cycling performance on a smaller but growing subset of synthesizable compositions.^14,15^ The frequency with which these two spaces address the same compositions makes the points of comparison easier to identify than in fields where computation and experiment study largely non-overlapping material sets.

Two features of how predictions are reported and cited make the relationship between calculation and measurement worth examining closely. The first concerns which prediction is foregrounded. Density functional theory (DFT) calculations of MXene anodes routinely produce two distinct capacity estimates for the same composition: one for the bare transition-metal-carbide surface and one for the surface bearing −O, −OH, or −F terminations.^9–11^ The terminated prediction corresponds to the form that synthesis produces; the bare prediction corresponds to a surface that is difficult to realize through current MXene synthesis routes.^16,17^ For Ti_3_C_2_, the terminated predictions of 67–130 mAh g^–1^ agree reasonably with measured values near 124 mAh g^–1^ at 1C, while the bare-surface prediction of 447 mAh g^–1^ is significantly higher.^8,18^ The difficulty is that the bare-surface value is most often propagated as the ″theoretical capacity″ of Ti_3_C_2_.^19,20^ A terminated material delivering close to its own correctly predicted capacity is thereby cast as underperforming against a figure that is realistically difficult to synthesize. The disconnect, in this case, is not between calculation and reality but between which calculation the literature chooses to carry forward.

The second feature of how predictions are reported concerns vocabulary. Computational and experimental studies both report capacity in mAh g^–1^, but the computational value is typically a rate-independent thermodynamic limit, whereas the experimental value is inseparable from the C-rate at which it was measured.^21^ The shared unit can therefore mask a comparison between quantities that are not directly equivalent. Structurally, real materials deviate from idealized computational models due to a mix of defect formation, surface oxidation, interlayer restacking, limited electrolyte accessibility, and evolving electrode architecture.^22^ Because these effects are rarely examined under coordinated computational and experimental conditions, it remains difficult to identify which assumptions or material features dominate the observed behavior. Closing this gap is not about making computations more complex in vacuum, nor is it about simply gathering more experimental data; instead, it is an opportunity to build a shared practice where both sides work in tandem to pinpoint exactly what drives performance in any given case.

## 3 Idealization Gap in Reported Capacities

The pattern is not unique to Ti_3_C_2_, but its visibility in other compositions depends on whether experimental measurements exist at all. The V_2_CO_2_ system illustrates how computational predictions move toward experimental agreement as the model captures more of the real structure. An early monolayer prediction exceeded the measured capacity by 28% (335 vs 260 mAh g^–1^), while a later multilayer calculation — accounting for the stacked geometry that the synthesized material actually adopts — reached 276 mAh g^–1^, within 6% at 1C.^15,23,24^ The improvement is instructive: it suggests that the gap between prediction and measurement narrows when the structural features of the operating material are accounted for. The difficulty is that this kind of agreement currently emerges case by case, through independent work rather than coordinated comparison, so the field has no systematic way of knowing in advance which predictions will hold.

Several methodological choices in current first-principles practice contribute to the gap (Table 1), and each is well-known in the broader DFT community even where its specific consequences for MXene-anode predictions remain underexplored. Standard DFT functionals, primarily the PBE generalized-gradient approximation, have established limitations for adsorption energetics on transition-metal surfaces, and whether these errors propagate quantitatively to Li–MXene binding energies has not been systematically benchmarked.^25^ Standard van der Waals (vdW) corrections (DFT-D2, DFT-D3, XDM and vdW-DF2) yield interlayer spacings and mechanical properties that vary substantially across schemes for MXene systems; recent benchmarking finds DFT-D3 and XDM closest to experiment while DFT-D2 overbinds and vdW-DF2 underbinds, with no single correction outperforming across all properties.^26^ The trend is consistent with the general flexibility of experiment-dependent dispersion corrections. DFT-D3 adjusts its dispersion coefficients according to the local coordination environment, while XDM derives them directly from the electron density, allowing both approaches to better account for chemically heterogeneous systems than the fixed-coefficient DFT-D2 scheme. Because MXene surfaces contain mixed terminations and diverse local coordination environments, such environment-sensitive treatments are expected to provide a more realistic description than approaches that assume uniform dispersion interactions.

**Table 1:** Standard computational protocols used in MXene-anode studies and the direction that would extend them toward operating conditions.

Method/Protocol	Captures	Omits (relevant to operating anode)	Extension toward operating regime	ref
DFT (PBE-GGA) adsorption energetics	Equilibrium binding on defined surfaces	GGA errors on transition-metal adsorption; vacuum reference excludes solvation	Benchmark functionals; reference to solvated Li^+^ rather than vacuum	^25^
van der Waals corrections (DFT-D2/DFT-D3/XDM/vdW-DF2)	Interlayer spacing, mechanical properties	Scheme-dependent spreads; choice rarely justified	Report sensitivity across schemes; use benchmarked correction (e.g., D3/XDM)	^26^
Convex-hull lithiation series	Thermodynamic reversible-capacity limit	Kinetically inaccessible configurations inflate capacity	Apply kinetic-accessibility screening routinely	^23^
AIMD (5–10 ps, NVT, 300 K)	Short-time structural stability	∼20 orders of magnitude short of a cycle; no SEI chemistry	ML interatomic potentials to reach ns−μs and reactive chemistry	^27^
Uniform single-termination surface model	Clean reference energetics	Real surfaces are mixed/heterogeneous −O/–OH/–F/–Cl	Configurational averaging/explicit patchy-surface modeling	^28^

The convex-hull construction commonly used to derive reversible capacity from a computed lithiation series is itself a thermodynamic argument, and one that includes Li configurations that may not form under kinetic cycling. For V_2_CO_2_, configurations with *x* > 1.5 (*x* is the number of Li atoms per formula unit in the bulk structure) were identified as kinetically inaccessible despite carrying negative formation energies and excluding them reduced the predicted capacity.^23^ Similar kinetic-accessibility analyses have not been routinely applied across the broader MXene-anode computational literature, leaving an unknown fraction of reported capacities sensitive to a correction that has been demonstrated to be significant where it has been applied.

## 4 Phenomena beyond Equilibrium Calculation

Standard computational protocols for MXene anodes describe well-defined idealized systems, and the predictions they generate are reliable for those systems. The operating anode, however, is a different system — one in which lithium arrives through a solvated state rather than from vacuum, where the surface is buried under a continuously evolving interphase, terminations vary across the electrode, and cycling proceeds out of equilibrium.^22,29,30^ The gap between predicted and operating behavior is not, in most cases, a matter of refinement; it is a matter of describing a system that excludes the physics responsible for cycling-life behavior.

### 4.1 Explicit Electrolyte Effects beyond Vacuum-Reference Calculations

Lithium insertion at an operating anode involves desolvation of Li^+^ from its carbonate solvation shell, an energy barrier that depends on solvent identity, salt concentration, and local electric field.^31^ Computational adsorption energies for MXene anodes are typically referenced to an isolated lithium atom or to bulk lithium metal, rather than to a lithium ion in its solvated state within the electrolyte, which omits the largest single contribution to the interfacial energetics and treats the rate-limiting step as if it were not present.^9,10^ Recent experimental work has shown that engineering Li^+^ desolvation at the Ti_3_C_2_ surface measurably boosts charge-storage performance, indicating that the energy landscape computation routinely omits has real consequences for what the cell delivers.^32^ Hybrid quantum-classical schemes that treat the MXene surface with DFT and the surrounding electrolyte with a polarizable force field already exist in adjacent computational electrochemistry, and adapting these schemes to MXene anodes would allow the desolvation energy to be computed directly.^33,34^

### 4.2 Time and Chemistry beyond Conventional *ab initio* Molecular Dynamics (AIMD)

The solid-electrolyte interphase (SEI) is usually absent from MXene-anode calculations, and its absence has more far-reaching consequences than the literature acknowledges. The SEI is not a passive film but a chemically and structurally evolving layer that mediates every Li^+^ transfer event, governs irreversible capacity loss in early cycles, and determines cycling lifetime.^31,35^ Modeling its formation requires reactive molecular dynamics (RMD) with explicit electrolyte decomposition pathways, capabilities that exist for related battery systems but have not been brought to MXene anodes at meaningful scale. The protocol that does appear in MXene-anode papers — AIMD run for 5–10 ps in the canonical ensemble at 300 K with a fully Li-loaded pristine surface — is sometimes offered as evidence of cycling stability.^27,36^ Five picoseconds is approximately 20 orders of magnitude shorter than a single charge–discharge cycle, and the simulation contains none of the chemistry that determines whether the material survives a thousand cycles. Machine-learning interatomic potentials (MLIP) trained on DFT energies and forces now make nanosecond-to-microsecond time scales accessible at near-DFT accuracy, and their extension to reactive electrolyte chemistry is a near-term direction the field can reasonably pursue.^12,37,38^

### 4.3 Real Surface Chemistry versus Idealized Terminations

Synthesized MXenes display mixed −O, −OH, −F, and increasingly −Cl terminations distributed heterogeneously across the surface, with the distribution depending on etchant chemistry, postsynthesis treatment, and oxidation history. Computational studies almost universally model uniform single-termination surfaces (−O_2_ or −F_2_), which produce adsorption energetics for a surface that cannot currently be synthesized in that form. Heterogeneous terminations create local variations in work function, binding strength, and ion accessibility, precisely the spatial variations that have been associated with nonuniform Li^+^ deposition in experimental work.^39^ Sampling realistic termination distributions in calculations, whether through configurational averaging or through explicit modeling of patchy surfaces informed by characterization, would let the calculation describe the surface the experiment actually probes.

### 4.4 Nonequilibrium Operations beyond Atomistic Equilibrium Models

Standard DFT and AIMD protocols are equilibrium calculations and have no mechanism for representing gradients, rate-dependent kinetics, or strain accumulation. The body of theoretical literature on MXene anodes therefore describes the material as it would behave at zero current, with no spatial gradients and no accumulated effects from prior cycling, a regime that is internally consistent but not the regime in which battery viability is determined. Multiscale frameworks that couple atomistic calculation to continuum representations of transport and strain are mature in adjacent battery-materials work, and their extension to MXene electrodes would bring practical cycling conditions inside the model.^40^

## 5 Building the Feedback Loop

Closing the gap requires building around the calculations the experimental constraint that disciplines them. Three coordinated directions — one structural, two methodological — would let the field do so.

### 5.1 Coordinated Theory-Experiment Studies as a Precondition

The most consequential change is the routine integration of computation and experiment within the same investigation, as demonstrated by adjacent battery-materials communities. Integrated battery testing and simulation frameworks have been applied to cell-level design and prototyping, with computational fluid dynamics (CFD) models refined against laboratory validation in iterative cycles.^41^ Recent work in alkali metal-ion battery materials has framed theoretical calculations as instrumental for experimentalists, with calculations specifically designed to guide electrode and electrolyte design and refined against measured values.^13^ Demonstrating an integrated theory–experiment workflow in the NiMoO_4_ system, density-functional predictions of capacity (203 mAh g^–1^) and the measured value (169 mAh g^–1^) were reported within the same study using matched conditions, and the calculation and the measurement informed each other rather than coexisting as parallel claims.^42^ Establishing this practice within MXene-anode research would transform computational predictions from standalone performance estimates into experimentally testable hypotheses.

### 5.2 Explicit Electrolyte Modeling as a Regime Extension

The first methodological direction addresses one of the principal sources of disconnect between calculated and operating energetics: the absence of the electrolyte. Rather than extending vacuum-reference adsorption energetics alone, future calculations should recover the desolvation contribution that governs lithium insertion under operating conditions through hybrid quantum-classical approaches.^43^ RMD with bond-breaking force fields and machine-learning potentials trained on reactive trajectories can extend this further to capture electrolyte decomposition and the early stages of SEI nucleation. Computational papers that report Li adsorption energies in vacuum should then be understood and cited as upper bounds on binding strength rather than as predictions of operating behavior — a reframing that does not diminish the value of the calculations but locates them correctly in the chain of inference between thermodynamic prediction and operating measurement. Coupled with the feedback loop, explicit electrolyte modeling becomes one of the routes by which calculation and measurement constrain each other on overlapping quantities rather than on quantities that look similar but are not.

### 5.3 MLIPs as a Time Scale Enabler

Once the feedback loop exists, calculations that reach the time scales of interfacial evolution become valuable rather than merely demonstrative. The approach has been demonstrated for silicon anodes during cycling, layered oxide cathodes under cell conditions, and solid electrolytes during ion transport.^44,45^ For MXene-electrolyte interfaces, the time scales relevant to SEI nucleation, termination rearrangement during cycling, and the slow restructuring that determines long-term capacity retention would be accounted for using machine learning-driven molecular dynamics (ML-MD). This adaptation to MXene anodes is a tractable extension rather than a fundamental innovation, and within a coordinated theory-experiment framework, the predictions ML-MD generates would be testable against operando measurement rather than offered as standalone forecasts. However, MLIP is only as representative as its training data: potentials trained predominantly on idealized, uniformly terminated DFT structures may extrapolate poorly to the mixed-termination, defected, and electrolyte-contacted surfaces of an operating anode, so the training set should deliberately sample the surface heterogeneity the experiment actually probes.^46^

## 6 Challenges and Perspectives

Building on that argument, this section sets out what we see as the principal open challenges, the research gaps that most constrain progress, and the directions, in both experiment and modeling methodology, that we believe the field should prioritize.

### 6.1 Idealized Surface Chemistry versus Real, Heterogeneous Terminations

The largest structural source of the prediction–measurement gap is that first-principles studies almost universally model uniform, simple surface chemistries, whereas synthesized MXenes carry mixed terminations distributed heterogeneously on the basal planes and/or edge sites. Because binding strength, work function, and ion accessibility all vary locally, an idealized-surface calculation predicts the energetics of a material that cannot currently be synthesized in that exact form. The discrepancies reported throughout this review therefore stem less from errors in the underlying physics than from applying idealized-surface predictions to a surface the model was never constructed to describe. What remains absent is not simply more sophisticated sampling of termination configurations, but experimentally constrained descriptions of surface chemistry that calculations and measurements can share. Progress will therefore depend on characterization-informed termination distributions rather than idealized termination states alone.

### 6.2 Reliability of Experimental Benchmarks

A recurring difficulty for any theory–experiment comparison is that the experimental literature is not always internally consistent enough to serve as a fixed benchmark. Reported capacities for nominally identical compositions vary substantially because synthesis route, electrode preparation, electrolyte choice, binder, mass loading, and testing protocol are often incompletely specified. This variability makes reproducibility uncertain and places a real limit on how sharply any prediction can be validated. We regard the establishment of standardized, fully reported experimental baselines, with synthesis and cycling conditions documented in enough detail to be modeled, as a precondition for meaningful benchmarking, and as an obligation shared by the experimental and computational communities rather than a problem for either alone.

### 6.3 Generalizability Across the M_n+1_X_n_T_x_ Family and beyond Lithium

Much of the quantitative understanding in this field is derived from Ti_3_C_2_T_*x*_, and a fair question is how far conclusions drawn from it extend. Our view is general across the MXene family and is not specific to titanium carbide MXenes. The *quantitative* heuristics, however, are composition-specific: magnitude of bare-versus-terminated discrepancy, preferred termination, and accessible lithium stoichiometry all depend on the M and X elements and n, and should not be transferred numerically from Ti_3_C_2_ to other members without recalculation. The same distinction applies across charge carriers. The conclusions here are developed for Li^+^ storage, but the underlying reasoning, i.e., desolvation, interphase formation, and termination heterogeneity, governing the gap applies with equal or greater force to Na^+^, K^+^, and multivalent (Mg^2+^, Ca^2+^, Al^3+^) systems, where larger ions, stronger solvation, and more complex interphases make idealized predictions even less directly comparable to measurement.

### 6.4 Priorities and Current Limits of Electrolyte and Interphase Modeling

Asked which electrolyte-related property should be prioritized in future computational work, we would rank Li^+^ desolvation energetics first. It is the primary energetic contribution absent from vacuum-referenced adsorption calculations; it is rate-limiting at the operating interface, and recent experiments show it measurably governs charge-storage performance at the Ti_3_C_2_ surface. SEI formation comes second but is substantially harder to reliably model; ion transport and decomposition pathways follow. As for the reliability of RMD for capturing electrolyte decomposition, the current reactive-force-field (ReaxFF) and reactive-MLIP approaches typically require extensive, system-specific reparameterization and validation before they can be trusted quantitatively, and transferable reactive descriptions of MXene–electrolyte chemistry are yet to exist.^47^

### 6.5 What Predictive Accuracy the Community Should Target

We propose that, once predictions are compared to properly reported baselines under matched conditions, agreement within roughly 10–20% of measured reversible capacity is a reasonable near-term target for a calculation that incorporates the appropriate surface termination and structural geometry, a level already demonstrated in isolated cases, such as the multilayer V_2_CO_2_ prediction that reached within 6% of the measured value once the stacked geometry was included.^23^

Taken together, the challenges above point to a coordinated program rather than a single breakthrough. On the experimental side, the field would benefit most from standardized, fully documented baselines and from operando characterization of termination chemistry and interphase evolution that can directly constrain models. On the modeling side, the priorities are realistic termination sampling, explicit and eventually reactive treatment of the electrolyte, and machine-learning potentials that reach cycling-relevant time scales while being trained on experimentally representative configurations.

## 7 Conclusion

The qualitative insights generated by a decade of computational MXene-anode work — the role of termination chemistry in setting binding strength and accessibility, the importance of metallic density of states at the Fermi level, the trade-off between binding strength and open-circuit voltage — survive the limitations described above because they are robust to the specific assumptions any individual calculation embeds. The quantitative predictions do not always survive in the same way because they are produced and cited without the experimental constraint that would let the field assess which of them to trust. Closing this gap is a matter of building the practice in which the two are evaluated against one another under shared experimental conditions. Coordinated theory-experiment studies as the structural precondition, explicit electrolyte modeling as the regime extension, and machine-learning interatomic potentials as the time scale enabler are three directions that, taken together, would let computational MXene-anode works answer the question this perspective has posed: what it takes for these predictions to become reliably predictive of experimental behavior. The next decade of MXene-anode work should be measured by how often computation and experiment describe the same physical system.

## References

[1] Gulavani V., John B., Sharma P., Yengantiwar A. (2025). Water-Based
Wonders: Exploring Recent Progress in Aqueous Lithium-Ion Batteries. Energy Fuels.

[2] Toki G. F. I., Hossain M. K., Rehman W. U., Manj R. Z. A., Wang L., Yang J. (2024). Recent progress and
challenges in silicon-based anode materials for
lithium-ion batteries. Industrial Chemistry
& Materials.

[3] Thakur A., Chandran B.S. N., Davidson K., Bedford A., Fang H., Im Y., Kanduri V., Wyatt B. C., Nemani S. K., Poliukhova V. (2023). Step-by-Step Guide for Synthesis and Delamination of Ti3C2T MXene. Small Methods.

[4] Lukatskaya M. R., Mashtalir O., Ren C. E., Dall’Agnese Y., Rozier P., Taberna P. L., Naguib M., Simon P., Barsoum M. W., Gogotsi Y. (2013). Cation Intercalation and High Volumetric
Capacitance of Two-Dimensional Titanium Carbide. Science.

[5] Khazaei M., Arai M., Sasaki T., Chung C.-Y., Venkataramanan N. S., Estili M., Sakka Y., Kawazoe Y. (2013). Novel Electronic and
Magnetic Properties of Two-Dimensional Transition Metal Carbides and
Nitrides. Adv. Funct. Mater..

[6] Anasori, B. ; Lukatskaya, M. R. ; Gogotsi, Y. 2D Metal Carbides and Nitrides (MXenes) for Energy Storage. In MXenes: From Discovery to Applications of Two-Dimensional Metal Carbides and Nitrides, 1st ed.; Gogotsi, Y. , Ed.; Jenny Stanford Publishing, 2023.

[7] Xie Y., Dall’Agnese Y., Naguib M., Gogotsi Y., Barsoum M. W., Zhuang H. L., Kent P. R. C. (2014). Prediction and Characterization of
MXene Nanosheet Anodes for Non-Lithium-Ion Batteries. ACS Nano.

[8] Ganesan M., Lee J. Y. (2025). Layered ion dynamics
and enhanced energy storage: VS_2_/MXene heterostructure
anodes revolutionizing Li-ion batteries. Nanoscale.

[9] Er D., Li J., Naguib M., Gogotsi Y., Shenoy V. B. (2014). Ti_3_C_2_ MXene
as a High Capacity Electrode Material for Metal (Li,
Na, K, Ca) Ion Batteries. ACS Appl. Mater. Interfaces.

[10] Tang Q., Zhou Z., Shen P. (2012). Are MXenes
Promising Anode Materials
for Li Ion Batteries? Computational Studies on Electronic Properties
and Li Storage Capability of Ti_3_C_2_ and Ti_3_C_2_×_2_ (X = F, OH) Monolayer. J. Am. Chem. Soc..

[11] Shetti N. P., Mishra A., Basu S., Aminabhavi T. M., Alodhayb A., Pandiaraj S. (2023). MXenes as
Li-Ion Battery Electrodes:
Progress and Outlook. Energy Fuels.

[12] Borah M., Wang Q., Moura S., Sauer D. U., Li W. (2024). Synergizing
physics and machine learning for advanced battery management. Communications Engineering.

[13] Zhang H., Liu X., Ji T., Ran J., Li Y., Shen Z., Wang X. (2026). Applications of theoretical calculations
in alkali metal-ion battery
investigation. Chin. Chem. Lett..

[14] Wang H., Jing Z., Liu H., Feng X., Meng G., Wu K., Cheng Y., Xiao B. (2020). A high-throughput assessment of the
adsorption capacity and Li-ion diffusion dynamics in Mo-based ordered
double-transition-metal MXenes as anode materials for fast-charging
LIBs. Nanoscale.

[15] Eames C., Islam M. S. (2014). Ion Intercalation
into Two-Dimensional Transition-Metal
Carbides: Global Screening for New High-Capacity Battery Materials. J. Am. Chem. Soc..

[16] Thangavelu H. H. S., Huang C., Chabanais F., Palisaitis J., Persson P. O. Å. (2026). A Review on MXene Terminations. Adv. Funct. Mater..

[17] Pranada E., Ngozichukwu B., Yoo R., Johnson D., Barteau M. A., Abdel-Wahab A., Djire A. (2025). Hydrogen Evolution and Oxygen Reduction
on OH/F-Terminated Titanium Nitride MXene Reveal the Role of the Surface
Termination Group in Electrocatalysis. ACS Catal..

[18] Sun D., Wang M., Li Z., Fan G., Fan L.-Z., Zhou A. (2014). Two-dimensional Ti_3_C_2_ as anode material for
Li-ion batteries. Electrochem. Commun..

[19] Wei C., Tian M., Fan Z., Yu L., Song Y., Yang X., Shi Z., Wang M., Yang R., Sun J. (2021). Concurrent realization of dendrite-free
anode and high-loading cathode
via 3D printed N-Ti_3_C_2_ MXene framework toward
advanced Li–S full batteries. Energy
Storage Materials.

[20] Yu Y.-X. (2016). Prediction
of Mobility, Enhanced Storage Capacity, and Volume Change during Sodiation
on Interlayer-Expanded Functionalized Ti_3_C_2_ MXene
Anode Materials for Sodium-Ion Batteries. J.
Phys. Chem. C.

[21] Heubner C., Schneider M., Michaelis A. (2020). Diffusion-Limited C-Rate: A Fundamental
Principle Quantifying the Intrinsic Limits of Li-Ion Batteries. Adv. Energy Mater..

[22] Ngozichukwu B., Pranada E., Dambo Y., Pranada J., Abhinav E., Djire A. (2026). Rethinking Performance Degradation in MXene Lithium-Ion Batteries
through Coupled Electrochemical Evolution. ACS
Energy Letters.

[23] Ashton M., Hennig R. G., Sinnott S. B. (2016). Computational
characterization of
lightweight multilayer MXene Li-ion battery anodes. Appl. Phys. Lett..

[24] Naguib M., Halim J., Lu J., Cook K. M., Hultman L., Gogotsi Y., Barsoum M. W. (2013). New Two-Dimensional Niobium and Vanadium
Carbides as Promising Materials for Li-Ion Batteries. J. Am. Chem. Soc..

[25] Cohen A. J., Mori-Sánchez P., Yang W. (2008). Insights into Current Limitations
of Density Functional Theory. Science.

[26] Rajashekhar
Vaibhava K. M., Vetrivel R., Singh V., Shanbhag G. V., Srikanth R. (2026). Investigation of van der Waals interactions in two-dimensional
MXenes via first-principles simulations. Phys.
Chem. Chem. Phys..

[27] Ahmed T., Piya A. A., Daula
Shamim S. U. (2024). Recent advances in Zr and Hf-based
MXenes and their hetero-structure as novel anode materials for Ca-ion
batteries: theoretical insights from DFT approach. Nanoscale Advances.

[28] Caffrey N. M. (2018). Effect
of mixed surface terminations on the structural and electrochemical
properties of two-dimensional Ti_3_C_2_T_x_ and V_2_CT_X_ MXenes multilayers. Nanoscale.

[29] Parker T., Zhang Y., Shevchuk K., Zhang T., Khokhar V., Kim Y.-H., Kadagishvili G., Bugallo D., Tanwar M., Davis B. (2025). In Situ
Raman and Fourier Transform Infrared Spectroscopy
Studies of MXene–Electrolyte Interfaces. ACS Nano.

[30] Ling J., Karuppiah C., Misnon I. I., Lee H. J., Yang C.-C., Jose R. (2024). In Situ Interphase Engineering for beyond Lithium-Ion Battery Technologies. Energy Fuels.

[31] Xu K. (2014). Electrolytes
and Interphases in Li-Ion Batteries and Beyond. Chem. Rev..

[32] Bo Z., Wang R., Wang B., Sunny S., Zhao Y., Ge K., Xu K., Song Y., Raymundo-Piñero E., Lin Z. (2025). Ion desolvation for boosting the charge storage performance
in Ti_3_C_2_ MXene electrode. Nat. Commun..

[33] Bi S., Knijff L., Lian X., van Hees A., Zhang C., Salanne M. (2024). Modeling of
Nanomaterials for Supercapacitors: Beyond
Carbon Electrodes. ACS Nano.

[34] Lin X., Tee S. R., Kent P. R. C., Searles D. J., Cummings P. T. (2024). Development
of Heteroatomic Constant Potential Method with Application to MXene-Based
Supercapacitors. J. Chem. Theory Comput..

[35] Peled E., Menkin S. (2017). Review—SEI: Past, Present and Future. J. Electrochem. Soc..

[36] Martins N. F., Laranjeira J. A. S., Aparicio-Huacarpuma B.
D., Ribeiro Junior L. A., Sambrano J. R. (2025). Computational Characterization of the Novel Ta_2_Se_2_C Transition Metal Carbo-Chalcogenide as Anode
Material for Li and Na-Ion Batteries. J. Phys.
Chem. C.

[37] Yao N., Chen X., Fu Z.-H., Zhang Q. (2022). Applying Classical,
Ab Initio, and Machine-Learning Molecular Dynamics Simulations to
the Liquid Electrolyte for Rechargeable Batteries. Chem. Rev..

[38] Friederich P., Häse F., Proppe J., Aspuru-Guzik A. (2021). Machine-learned
potentials for next-generation matter simulations. Nat. Mater..

[39] Yang D., Zhao C., Lian R., Yang L., Wang Y., Gao Y., Xiao X., Gogotsi Y., Wang X., Chen G. (2021). Mechanisms of the Planar
Growth of Lithium Metal Enabled by the 2D
Lattice Confinement from a Ti_3_C_2_T_x_ MXene Intermediate Layer. Adv. Funct. Mater..

[40] Franco A. A., Rucci A., Brandell D., Frayret C., Gaberscek M., Jankowski P., Johansson P. (2019). Boosting Rechargeable Batteries R&
D by Multiscale Modeling: Myth or Reality?. Chem. Rev..

[41] Liaw B. Y., Bethune K. P., Yang X. G. (2002). Advanced integrated
battery testing
and simulation. J. Power Sources.

[42] Sawant D. S., Gaikwad S. V., Fulari A. V., Govindasamy M., Kulkarni S. B., Dubal D. P., Lohar G. M. (2025). Theoretical Specific
Capacity and Metal Ion Diffusion Pathway of NiMoO_4_Microspheres
for Hybrid Supercapacitors. Small.

[43] Naserifar S., Chen Y., Kwon S., Xiao H., Goddard W. A. (2021). Artificial
Intelligence and QM/MM with a Polarizable Reactive Force Field for
Next-Generation Electrocatalysts. Matter.

[44] Zhang B., Ma T., He N., Wang C., Tan H., Han J., Liu Y. (2025). Machine learning driven insights into lithiation mechanisms at the
silicon-graphite interface within composite electrode. Acta Mater..

[45] Zhao D., Guan Y., Wu Z., Zhang L. (2023). Thermal Stability and
Mechanical Properties of Hollow Si Nanowires from Atomic Modeling
Combined with a Machine-Learning Prediction for Application as Li-Ion
Battery Anodes. ACS Applied Nano Materials.

[46] Xie W., Han Y., Wu C., Hu P. (2026). Smarter Data: Rethinking Data Generation
for Machine Learning Potentials in Heterogeneous Catalysis. JACS Au.

[47] De
Angelis P., Cappabianca R., Fasano M., Asinari P., Chiavazzo E. (2024). Enhancing ReaxFF for molecular dynamics simulations
of lithium-ion batteries: an interactive reparameterization protocol. Sci. Rep..

